# Free-standing graphene/bismuth vanadate monolith composite as a binder-free electrode for symmetrical supercapacitors

**DOI:** 10.1039/c8ra04200d

**Published:** 2018-07-10

**Authors:** Lingjuan Deng, Jiahuan Liu, Zhanying Ma, Guang Fan, Zong-huai Liu

**Affiliations:** College of Chemistry and Chemical Engineering, Xianyang Normal University Xianyang Shaanxi 712000 P. R. China denglingjuan@163.com +86-29-33720704; Key Laboratory of Applied Surface and Colloid Chemistry, Shaanxi Normal University, Ministry of Education Xi’an 710062 P. R. China; School of Materials Science and Engineering, Shaanxi Normal University Xi’an 710062 P. R. China

## Abstract

Preparation of new types of electrode material is of great importance to supercapacitors. Herein, a graphene/bismuth vanadate (GR/BiVO_4_) free-standing monolith composite has been prepared *via* a hydrothermal process. Flexible GR sheets act as a skeleton in the GR/BiVO_4_ monolith composites. When used as a binder-free electrode in a three-electrode system, the GR/BiVO_4_ composite electrode can provide an impressive specific capacitance of 479 F g^−1^ in a potential window of −1.1 to 0.7 V *vs.* SCE at a current density of 5 A g^−1^. A symmetrical supercapacitor cell which can be reversibly charged–discharged at a cell voltage of 1.6 V has been assembled based on this GR/BiVO_4_ monolith composite. The symmetrical capacitor can deliver an energy density of 45.69 W h kg^−1^ at a power density of 800 W kg^−1^. Moreover, it ensures rapid energy delivery of 10.75 W h kg^−1^ with a power density of 40 kW kg^−1^.

## Abstract 2

A symmetrical supercapacitor with a high energy density has been assembled based on a free-standing GR/BiVO_4_ monolith composite.

## Graphical abstract

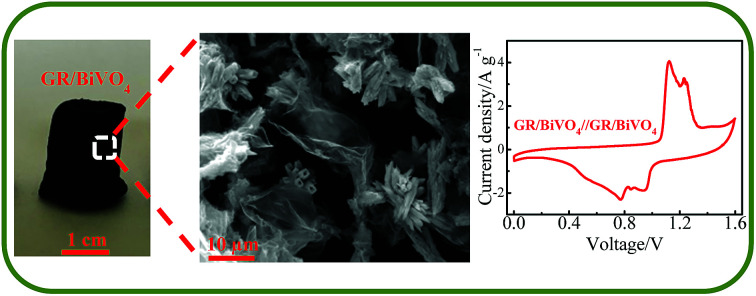

## 1. Introduction

Energy storage and conversion have played big roles in the history of human civilization. Nowadays, nobody can imagine how to live without electrochemical energy storage systems such as mobile phones, iPads, personal computers, and so on. Supercapacitors, also called electrochemical capacitors, hold great promise for energy storage and power supply due to their high power densities, long cycle lives, high efficiencies, environmental friendliness, safety and so on.^1^ It has been demonstrated that supercapacitors can be used as hybrid power sources, backup power sources, starting power for fuel cells, and for burst-power generation in electronic devices.^1^ However, the limited energy densities of supercapacitors hinder their applications for energy storage. As is well known, electrode materials are the key factor affecting the capacitive properties of supercapacitors. So preparing electrode materials with high capacitances, long cycling lives and high working voltages is desirable for supercapacitors with high energy densities.

Carbon materials, metal oxides and conducting polymers are the classic electrode materials for supercapacitors.^2^ Carbon materials (active carbon, graphene, carbon nanotubes, and so on) are representative of electrical double layer capacitor (EDLC) electrode materials, and these only show pure physical charge accumulation rather than electrochemical reactions occurring at the electrode/electrolyte interface during charge–discharge processes.^3^ Metal oxides (RuO_2_, MnO_2_, V_2_O_5_, Mn_3_O_4_, NiO, *etc.*) and conducting polymers (polyaniline and polypyrrole) are typical pseudocapacitor electrode materials, and they store charges mainly through electrochemical reactions during charge–discharge processes.^4^ Pseudocapacitors have been proven to supply much higher specific capacitances and energy densities than EDLCs.^1^ Exploiting new kinds of pseudocapacitor electrodes remains of great importance to supercapacitors.

BiVO_4_ has been widely used as a pigment in our daily lives (*e.g.* in traffic signs) due to its non-toxicity and bright yellow color. With a band-gap of 2.4 eV and chemical stability in aqueous solutions under irradiation, monoclinic BiVO_4_ has been widely used as a photocatalyst and a photoelectrode.^5–7^ Most recently, it has been demonstrated that BiVO_4_ can act as an electrode material for supercapacitors owing to its excellent physicochemical properties and stability.^8–13^ However, BiVO_4_ possesses the common fault of poor electrical conductivity as a metal oxide, which hampers capacitance retention and rate capability during fast charge–discharge processes.^13^ Therefore, improving electrical conductivity is be crucial for the use of BiVO_4_-based materials in supercapacitors.

Graphene (GR) is believed to be a prime candidate for an electrode material for EDLCs due to the fact that it is one-atom thick, has excellent mechanical flexibility, superior electrical conductivity and so on.^14^ GR can give an encouraging EDLC of 550 F g^−1^ if all of the theoretical specific surface area can be fully utilized. Generally, pure GR possesses unsatisfactory capacitive properties resulting from the partial sacrifice of specific surface area during the formation of irreversible agglomerates.^15^ For this reason, GR is usually used to form composites with pseudocapacitor electrode materials to achieve excellent capacitive properties.^16–19^

Although there are some reports based on GR/BiVO_4_ composites for supercapacitors, all of these materials are in the form of powders, and thus the conductive and binder additives must be added when the electrodes are prepared. Ultimately, the specific capacitances of BiVO_4_ electrodes would be reduced due to the increasing of the electrode masses. Binder-free electrodes based on BiVO_4_ for supercapacitors are rarely reported. In this work, a novel graphene/bismuth vanadate (GR/BiVO_4_) free-standing monolith composite has been prepared by hydrothermal technology. GR nanosheets play a big part in the GR/BiVO_4_ monolith composites, not just as a skeleton, but also as a conductive agent. The GR/BiVO_4_ composites can be used as binder-free electrode materials for supercapacitors and give a high specific capacitance of 479 F g^−1^ at a current density of 5 A g^−1^ in 2.0 mol L^−1^ NaOH solution. A symmetrical supercapacitor which can be reversibly charged–discharged at a cell voltage of 1.6 V has been assembled. The symmetrical capacitor can deliver an energy density of 10.75 W h kg^−1^ at a power density of 40 kW kg^−1^ together with good cycling stability.

## 2. Experimental

### 2.1. Materials preparation

Graphite oxide (GO) was fabricated using crude flake graphite (Qingdao Aoke Co.) as precursor by the classic Hummers method with some modifications.^20^ A GO homogeneous aqueous dispersion (4 mg mL^−1^) was obtained by ultrasonicating a mixture of 400 mg as-prepared GO powders and 100 mL distilled water for 3 hours.

The GR/BiVO_4_ free-standing monolith composite was prepared as follows: firstly, 1 mL Bi(NO_3_)_3_ solution (0.25 mmol Bi(NO_3_)_3_·5H_2_O dissolved in 1 mL glacial acetic acid) was added to the GO dispersion (35 mL, 4 mg mL^−1^) drop by drop. Secondly, 1 mL NH_4_VO_3_ aqueous solution (0.25 mmol NH_4_VO_3_ dissolved in 1 mL boiling H_2_O) was added dropwise into the above mixture, and then ammonium hydroxide was added to make the pH of the solution neutral. After violently stirring for 10 min, the bright yellow dispersion was transferred into an autoclave and heated at 180 °C for 12 h. Finally, the resulting free-standing monolith was removed, and dialyzed with distilled water to a neutral pH. BiVO_4_ powders were prepared by the same process without adding the GO dispersion.

The GR free-standing monolith was prepared by heating the GO dispersion (35 mL, 4 mg mL^−1^) at 180 °C for 12 h.

### 2.2. Characterization

X-ray diffraction (XRD) measurements were carried out using an Ultima IV diffractometer. A Quanta 600 FEG field emission scanning electron microscope (FESEM) and a transmission electron microscope (TEM) (JEM2010-HR) were used to observe the morphologies of the obtained materials. A Micromeritics ASAP 2020 nitrogen adsorption apparatus was used to investigate the BET surface areas and porous properties following degassing at 120 °C for 3 h below 10^−3^ mmHg. Cyclic voltammetry (CV), galvanostatic charge–discharge and electrochemical impedance spectroscopy (EIS) measurements of the different electrodes were carried out using a CHI660E electrochemical workstation. Cycle stability measurements were measured using a battery testing system (LAND, ModelCT2001A).

### 2.3. Electrochemical measurements

The GR/BiVO_4_ and GR monolith electrodes used for electrochemical testing were prepared as follows: slices of the materials with thicknesses of ∼1 mm (about 3 mg) were first cut from the purified monolith, and then were sandwiched between two stainless steel net layers (500 mesh, one piece: 1.5 cm × 1.5 cm, and the other: 1.5 cm × 10 cm) under a pressure of ∼6 MPa for 1 min. The mass of the electrode materials in the electrode was determined by calculating the weight difference before and after drying at 110 °C for 12 h.

BiVO_4_ electrodes were prepared as follows: BiVO_4_ powder, acetylene black and polyvinylidene fluoride (0.02 g mL^−1^, in *N*-methyl-ketopyrrolidine) (75 : 20 : 5 by weight) were mixed to obtain a slurry. Then the slurry was sandwiched between two stainless steel net layers (500 mesh, one piece: 1.5 cm × 1.5 cm, and the other piece: 1.5 cm × 10 cm) under a pressure of ∼6 MPa for 1 min. The mass of the active materials in the electrode was determined by calculating the weight difference before and after drying at 110 °C for 12 h. The loading mass of the active material was about 5 mg.

The electrochemical properties of the electrodes were measured in a three-electrode system which contained a platinum counter electrode, a saturated calomel electrode (SCE) reference electrode and 2.0 mol L^−1^ NaOH aqueous electrolyte, respectively.

Symmetrical supercapacitors were assembled as follows: two GR/BiVO_4_ composite monolith electrodes with the same mass were separated by a glass paper fiber which had been saturated in 2.0 mol L^−1^ NaOH electrolyte without the removal of oxygen from the solution. The GR/BiVO_4_//GR/BiVO_4_ symmetrical supercapacitor cells were assembled by placing the electrode and glass fiber layers in stainless steel clamps sandwiched between two nickel foam current collectors.

The specific capacitances *C* (F g^−1^) of the electrode in the three-electrode system and the symmetrical supercapacitors, and the energy densities *E* (W h kg^−1^) and power densities *P* (W kg^−1^) of the supercapacitors were calculated from the galvanostatic charge–discharge results as follows:1
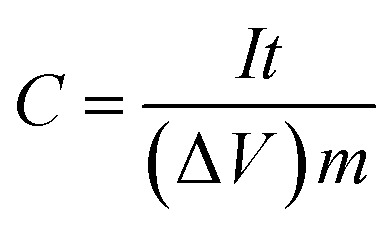
2
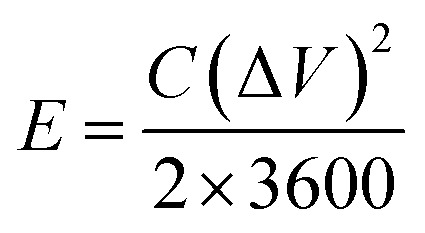
3
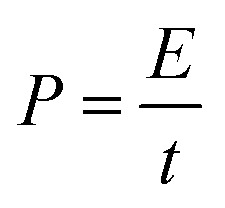
where Δ*V* = (*V*_max_ − *V*_min_), and *V*_max_ and *V*_min_ are the potentials at the end of charge and discharge, respectively. *m* is the active mass of the electrode or symmetrical supercapacitor (kg), *I* is the constant current (A) and *t* is the discharge time (s).

## 3. Results and discussion

**Fig. 1:**
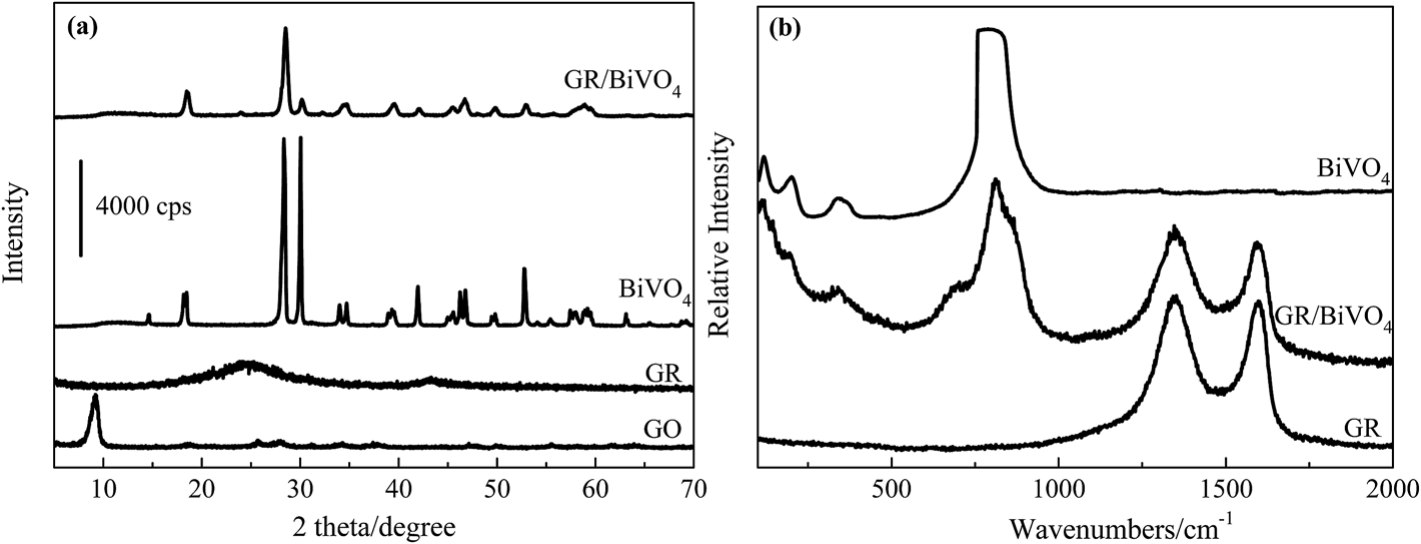
XRD patterns (a) and Raman spectra (b) of BiVO_4_, the GR/BiVO_4_ composite and GR, respectively.

Fig. 1a depicts the XRD patterns of GO, GR, BiVO_4_ and the GR/BiVO_4_ composite, respectively. GR only exhibits a broad peak centered at 24.1° indicating the formation of a poorly ordered graphite-like material.^21^ The XRD pattern of the as-prepared BiVO_4_ powder gives a crystalline monoclinic phase with lattice constants *a* = 0.5185 nm, *b* = 1.1713 nm and *c* = 0.5102 nm which are in good agreement with literature values (JCPDS card no. 014-0688).^13^ The pattern of the GR/BiVO_4_ composite also exhibits the diffraction peaks of monoclinic BiVO_4_. The characteristic diffraction peak of GR in the XRD pattern of the GR/BiVO_4_ composite disappears suggesting that the GR nanosheets are well dispersed in the composite. There are four well-indexed peaks located at 114, 200, 345 and 810 cm^−1^ for both BiVO_4_ and the GR/BiVO_4_ composite in the Raman spectra (Fig. 1b), which clearly demonstrate that BiVO_4_ was obtained in the final products.^22^ Two peaks at 1349 and 1596 cm^−1^ in the Raman spectra provide the evidence that GR remains in the GR/BiVO_4_ composite.^23^

**Fig. 2:**
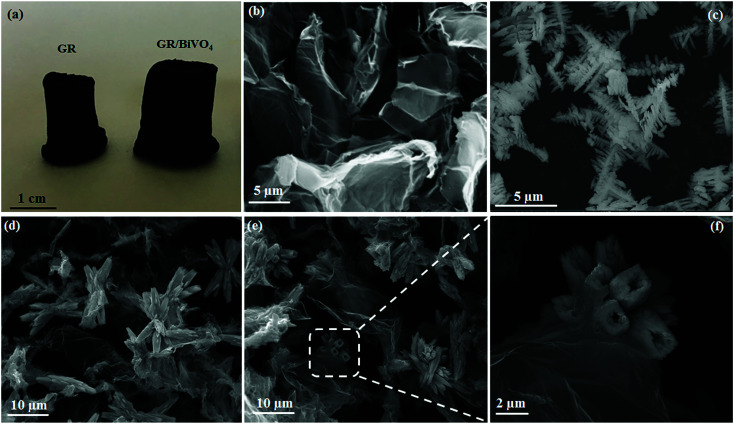
Digital photos (a) of the free-standing GR and GR/BiVO_4_ monoliths. FESEM images of GR (b), BiVO_4_ (c) and the GR/BiVO_4_ composite (d–f), respectively.

From the digital photos of GR and the GR/BiVO_4_ monolith (Fig. 2a), we can see that the size of the GR/BiVO_4_ monolith is greater than that of the GR monolith although the same reaction vessel was used. Pure BiVO_4_ was obtained in the form of a powder compared with the monolith-like GR/BiVO_4_ composite, suggesting that the GR nanosheets act as a skeleton while the BiVO_4_ particles act as spacers to pillar GR, and ultimately give a larger sized product. FESEM images of GR, BiVO_4_ and the GR/BiVO_4_ composite are shown in Fig. 2b–f. The GR monolith exhibits a loose and porous morphology due to the random stacking of GR nanosheets. BiVO_4_ gives a special spruce dendritic-like morphology formed by a number of branches with length sizes in the range 0.3–1 μm, and the full unit is supported by a unique backbone with a length of about 5 μm. It is reported that such a structure can supply high porosity which favors the access of electrolyte ions.^13^

A close look at Fig. 2d clearly reveals that the GR nanosheets insert randomly into the BiVO_4_ particles. BiVO_4_ in the GR/BiVO_4_ composite give a totally different morphology compared with that of pure BiVO_4_ particles. It seems that the backbones of the spruce dendrites become shorter and the branches grow longer and thicker. During the hydrothermal reaction, the growth of BiVO_4_ particles was limited by steric hindrance due to the presence of GO nanosheets. In order to achieve a minimal total specific surface free energy, BiVO_4_ particles have to adjust their structures and finally attain a flower-like morphology. Most interestingly, BiVO_4_ in the composite displays hollow branch tips with a wall thickness of 1 μm (Fig. 2f), and such a morphology is beneficial for the accommodation of electrolyte ions.

**Fig. 3:**
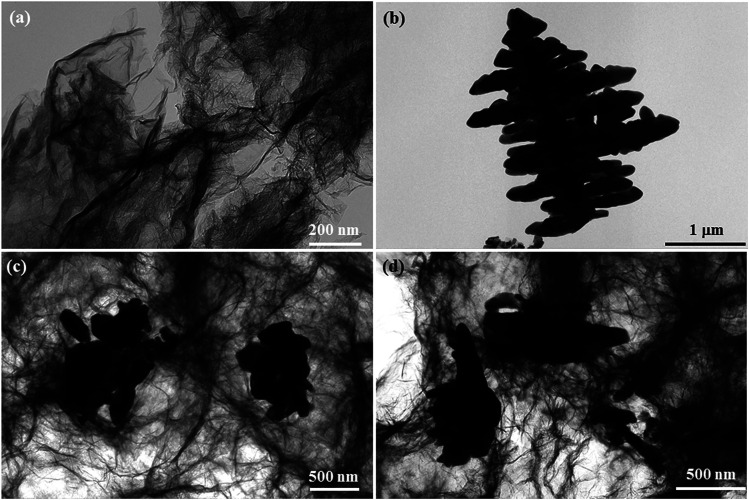
TEM images of GR (a), BiVO_4_ (b) and the GR/BiVO_4_ composite (c, d), respectively.

TEM was employed to monitor the microstructures of GR, BiVO_4_ and the GR/BiVO_4_ composites. Fig. 3a shows a silk-like morphology which is composed of ultrathin GR nanosheets with sizes of several hundreds of micrometers. BiVO_4_ presents a spruce dendritic-like morphology surrounded with many branches, and the sizes are consistent with those from the FESEM results. From Fig. 3c and d, we can see that the BiVO_4_ particles with different sizes randomly exist on the GR nanosheets. Just owing to the existence of BiVO_4_ particles, the reassembling of GR could be prevented to some degree. Generally, GR can improve the electrical performance of the composite and also facilitate fast transportation of electrons during electrochemical reactions. It is expected that the GR/BiVO_4_ composite could give an excellent electrochemical performance for supercapacitors.

**Fig. 4:**
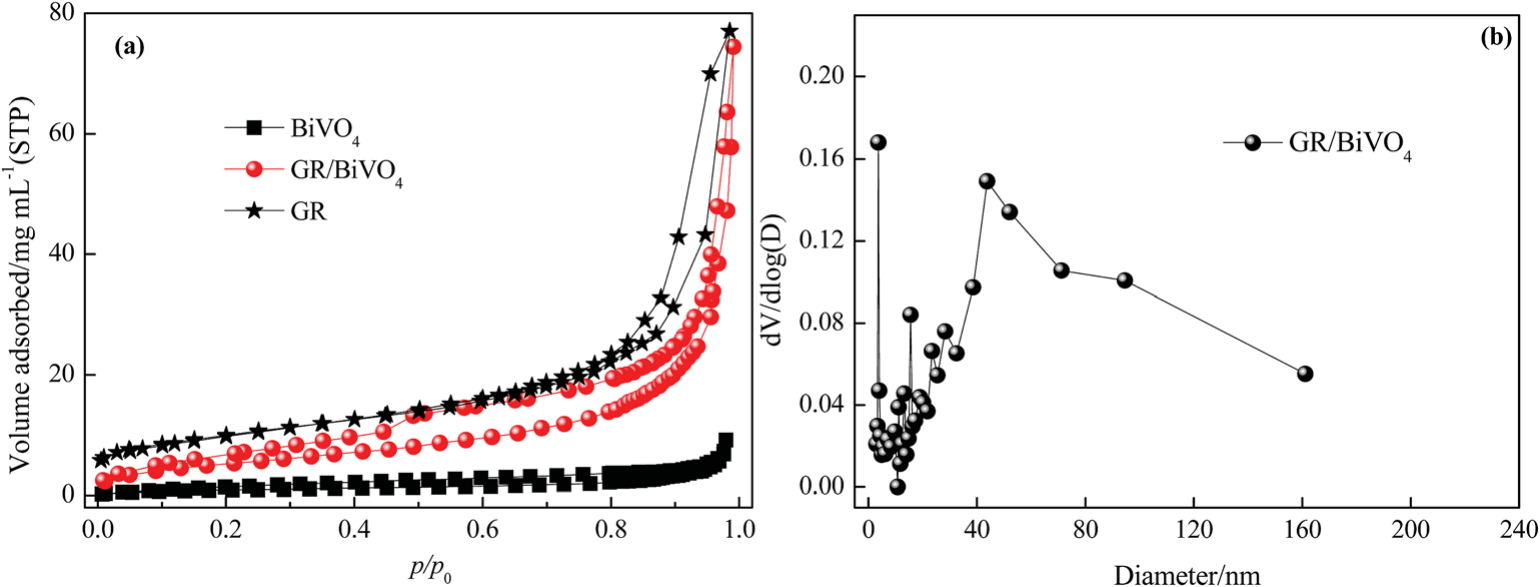
(a) N_2_ adsorption/desorption isotherms of GR, BiVO_4_ and the GR/BiVO_4_ composite, respectively. (b) Pore size distribution of the GR/BiVO_4_ composite.

The porous nature of the GR/BiVO_4_ monolith was studied with N_2_ adsorption and desorption tests (Fig. 4a). The GR/BiVO_4_ monolith exhibits a type IV isotherm with a H3 hysteresis loop at high relative pressure, indicating the existence of plentiful mesopores.^24^ The BET method reveals a specific surface area of 36 m^2^ g^−1^ for the GR/BiVO_4_ monolith, which is between that of the GR monolith (77.5 m^2^ g^−1^) and BiVO_4_ powder (3.8 m^2^ g^−1^). The pores constructed by the GR nanosheets and BiVO_4_ particles are mainly mesopores with a pore size distribution of 3–50 nm (calculated from the desorption data using the Barrette–Joyner–Halenda model) and an average pore size of about 23.5 nm (Fig. 4b). Because the hydrated ions can easily access the exterior and interior pore surfaces during the charge–discharge process, this GR/BiVO_4_ monolith with a moderate specific surface area and appropriate pore size could improve both the main pseudocapacitance of BiVO_4_ and the EDLC capacitance of GR.

**Fig. 5:**
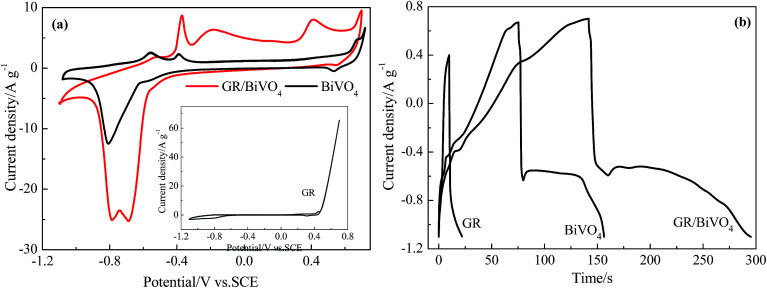
Electrochemical capacitive properties of GR, BiVO_4_ and GR/BiVO_4_ composite electrodes measured using a three-electrode system: (a) CV curves at scan rate of 5 mV s^−1^ and (b) galvanostatic discharge profiles at a current density of 5 A g^−1^.

The electrochemical behavior of BiVO_4_, the GR/BiVO_4_ composite and GR was studied by performing CV, galvanostatic charge–discharge and electrochemical impedance spectroscopy (EIS) tests, respectively. CV measurements were performed at a scan rate of 5 mV s^−1^ in the potential window of −1.1 V to 0.7 V *vs.* SCE (Fig. 5a). The GR electrode exhibits a sharp current increase when the potential is over 0.4 V *vs.* SCE, indicating an unsuitable potential window. For the BiVO_4_ electrode, a single peak with a high current around −0.72 V *vs.* SCE for the reduction was obtained which was assigned to the reduction of Bi^3+^ to Bi^0^, on the other side, the two main anodic peaks were obtained at −0.62 V and −0.43 V for the oxidation of Bi^0^ to Bi^+^ and Bi^+^ to Bi^3+^, respectively.^13,25^ The small peak located between 0.42 and 0.55 V *vs.* SCE in Fig. 5a may result from Na^+^ insertion and de-insertion reactions, and this phenomenon has been previously reported for vanadium oxide.^26,27^ The CV profile of the GR/BiVO_4_ electrode matched well with that of the BiVO_4_ electrode demonstrating that the redox peaks are due to BiVO_4_ and the presence of GR does not affect its electrochemical response. Such behavior should be regarded as battery performance according to the opinion of Patrice Simon.^28^ Due to the introduction of GR, the electrical conductivity of the GR/BiVO_4_ composite is much improved and thus a greater current density compared to the pure BiVO_4_ electrode is obtained in the same potential window. This phenomenon is similar to that of a GR/BiVO_4_ composite photocatalyst,^29^ in which the photogenerated electrons from BiVO_4_ nanoparticles could fast transfer to GR nanosheets and therefore an effective charge separation combined with a higher photocatalytic activity was finally obtained.

The specific capacitances of BiVO_4_, GR/BiVO_4_ and GR were evaluated with galvanostatic charge–discharge measurements and are presented in Fig. 5b. The galvanostatic charge–discharge profile of BiVO_4_ is non-symmetric with two different regions (a steep voltage drop and a prolonged plateau of voltage in the total potential range), suggesting the pseudocapacitive nature of this material for charge storage applications. The sudden steep drop within the first few seconds during the discharge section indicates the big internal resistance of BiVO_4_. The prolonged plateau of voltage output is due to the involvement of a quasi-Faradaic process in the BiVO_4_ electrode. The GR/BiVO_4_ electrode gives a similar galvanostatic charge–discharge curve to that of the BiVO_4_ electrode in the potential window of −1.1 V to 0.7 V *vs.* SCE. Using eqn (1), the specific capacitance value of GR/BiVO_4_ was found to be 479 F g^−1^ at 5 A g^−1^ much higher than that of the BiVO_4_ electrode (224 F g^−1^ at 5 A g^−1^) or the GR electrode (40 F g^−1^ at 5 A g^−1^ in the potential window of −0.7 to 0.4 V *vs.* SCE).

**Fig. 6:**
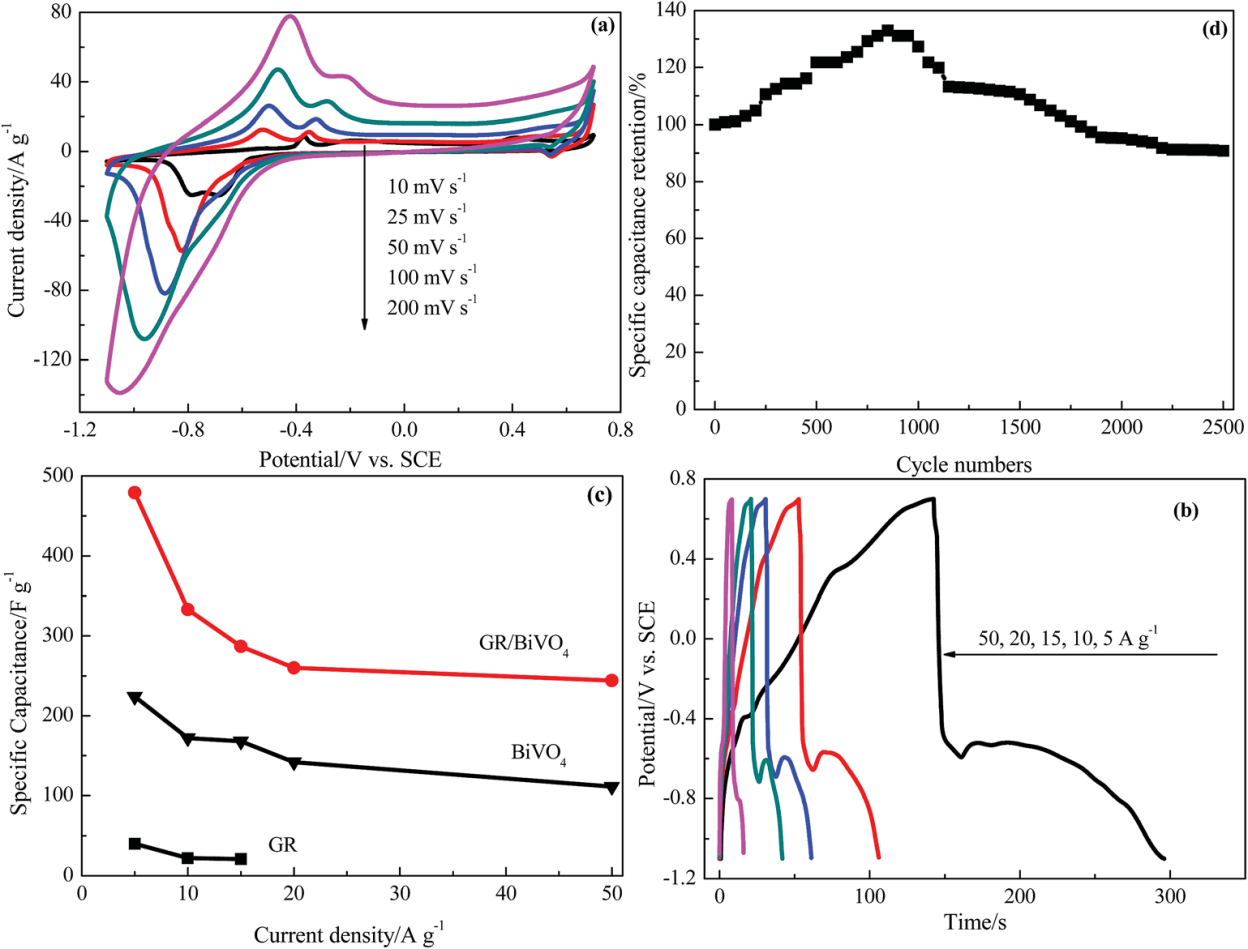
Electrochemical capacitive properties of the GR/BiVO_4_ composite electrode in a three-electrode system: (a) CV curves at different rates, (b) galvanostatic discharge profiles at different current densities, (c) specific capacitances at different current densities and (d) the variation of specific capacitance with cycle number.

Fig. 6a shows the CV curves of the GR/BiVO_4_ electrode at different scan rates in the potential window of −1.1 to 0.7 V *vs.* SCE in a three-electrode system. The shape of the CV curve of GR/BiVO_4_ is maintained even at a high scan rate of 200 mV s^−1^, which further confirms the improved pseudocapacitive behavior and the fast diffusion of ions into the BiVO_4_. Along with increasing scan rate, the CV curves of the GR/BiVO_4_ electrode show the oxidation peaks shifting positively while the reduction ones shift negatively resulting from the internal resistance, and this phenomenon has been widely reported for pseudocapacitor electrode materials.^12,30–32^

Fig. 6b displays the galvanostatic charge–discharge curves of the GR/BiVO_4_ electrode at various current densities of 5–50 A g^−1^ within the potential window of −1.1–0.7 V *vs.* SCE. The discharge times get shorter as the current density increases. When the current density is 50 A g^−1^, the platform in the discharge curve is obscured due to insufficient access time for the electrolyte ions penetrating into the GR/BiVO_4_ electrode. Fig. 6c gives the specific capacitances of the BiVO_4_, GR/BiVO_4_ and GR electrodes at different current densities. A higher specific capacitance (244 F g^−1^) for the GR/BiVO_4_ electrode is observed compared with that of the BiVO_4_ electrode (111 F g^−1^) at a current density of 50 A g^−1^. The GR/BiVO_4_ electrode exhibits a capacitance retention of nearly 51% even when the current density increased by 10 times. In our previous work, we studied the capacitive properties of a RuO_2_/GR composite in H_2_SO_4_ electrolyte, and the results showed that this RuO_2_/GR composite electrode gave a specific capacitance of 479 F g^−1^ at a current density of 0.25 A g^−1^ in potential window of 0–1.0 V *vs.* SCE.^33^ The GR/BiVO_4_ electrode exhibits a comparable specific capacitance value to that of this RuO_2_-based electrode, but a broader potential window is obtained. The specific capacitance of the GR/BiVO_4_ electrode is also higher than that of most previously used pseudocapacitor electrode materials.^34–37^ It seems that the specific capacitance values of the GR/BiVO_4_ electrode are smaller than those of LDHs (often reported as about 1000 F g^−1^),^38–40^ but a wide potential window (1.8 V) compared to that of LDHs (normally 0.5 V) is obtained.

After 2500 consecutive charge–discharge cycles at a current density of 10 A g^−1^, the specific capacitance of the GR/BiVO_4_ electrode was 302 F g^−1^, giving a capacitance retention of 91% (Fig. 5d). The obvious capacitance growth in the initial 1000 cycles could be ascribed to the electrode/electrolyte interface through the wetting process.

**Fig. 7:**
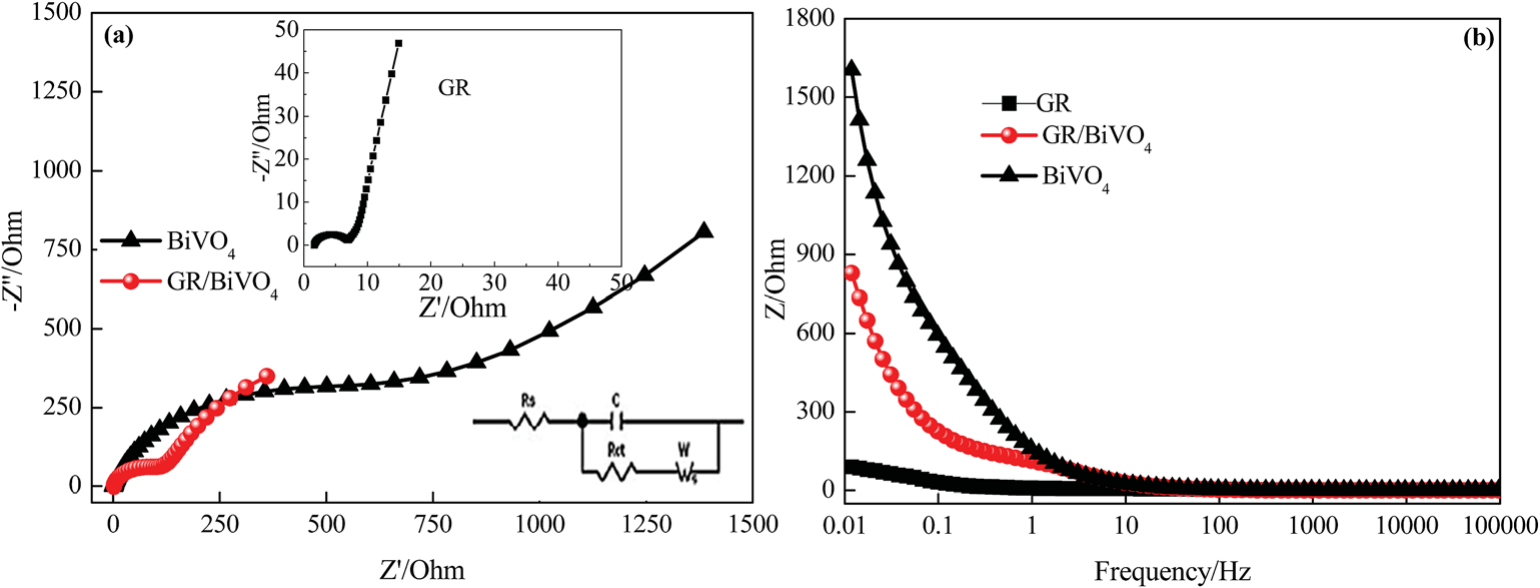
EIS tests of the GR, BiVO_4_ and GR/BiVO_4_ composite electrodes, respectively. (a) Nyquist plots measured within the frequency range of 100 kHz to 0.01 Hz and (b) plot of total impedance *versus* frequency.

The ion diffusion kinetics within the prepared electrodes were monitored by EIS. Fig. 7a shows the Nyquist plots of the BiVO_4_, GR and GR/BiVO_4_ electrodes within the frequency range 10 mHz to 100 kHz at a signal voltage of 5 mV. At very high frequency, the real part of resistance (*Z*′) of the intercept of the plot with the real axis, represents the equivalent series resistance (*R*_s_) provided by the ionic resistance of the electrolyte, the intrinsic resistance of the active materials and the contact resistance with the current collector. At medium–high frequencies, a distinct semicircular loop is observed, which represents charge transfer resistance (*R*_ct_) at the interface between the electrolytes and electrode. After fitting the EIS spectra through the equivalent circuit diagram (inset in Fig. 7a), the *R*_ct_ values are 4.7, 541.1 and 84.8 Ω for the GR, BiVO_4_ and GR/BiVO_4_ electrodes, respectively. The BiVO_4_ electrode shows the biggest *R*_ct_ value resulting from poor electrical conductivity. The GR/BiVO_4_ electrode, however, exhibits a smaller *R*_ct_ value than that of the BiVO_4_ electrode, indicating that the electrical conductivity of BiVO_4_ electrodes can be improved by adding GR.

The relationship between the total impedance and the frequency for the BiVO_4_, GR and GR/BiVO_4_ electrodes is shown in Fig. 7b, respectively. At low frequency, electrolyte ions could reach all of the pores with different sizes, and therefore the alternating current signal must penetrate through different depths thereby giving a big resistance. However, the electrolyte ions can only reach the rims of the pores to give small resistance values at high frequency.^41^ As exhibited in Fig. 7b, the BiVO_4_ electrode shows appalling resistance below 1 Hz, suggesting a disappointing electrical conductivity. The introduction of GR, fortunately, could improve its electrical conductivity, with the resistance of the GR/BiVO_4_ electrode located between the GR and BiVO_4_ electrodes.

**Fig. 8:**
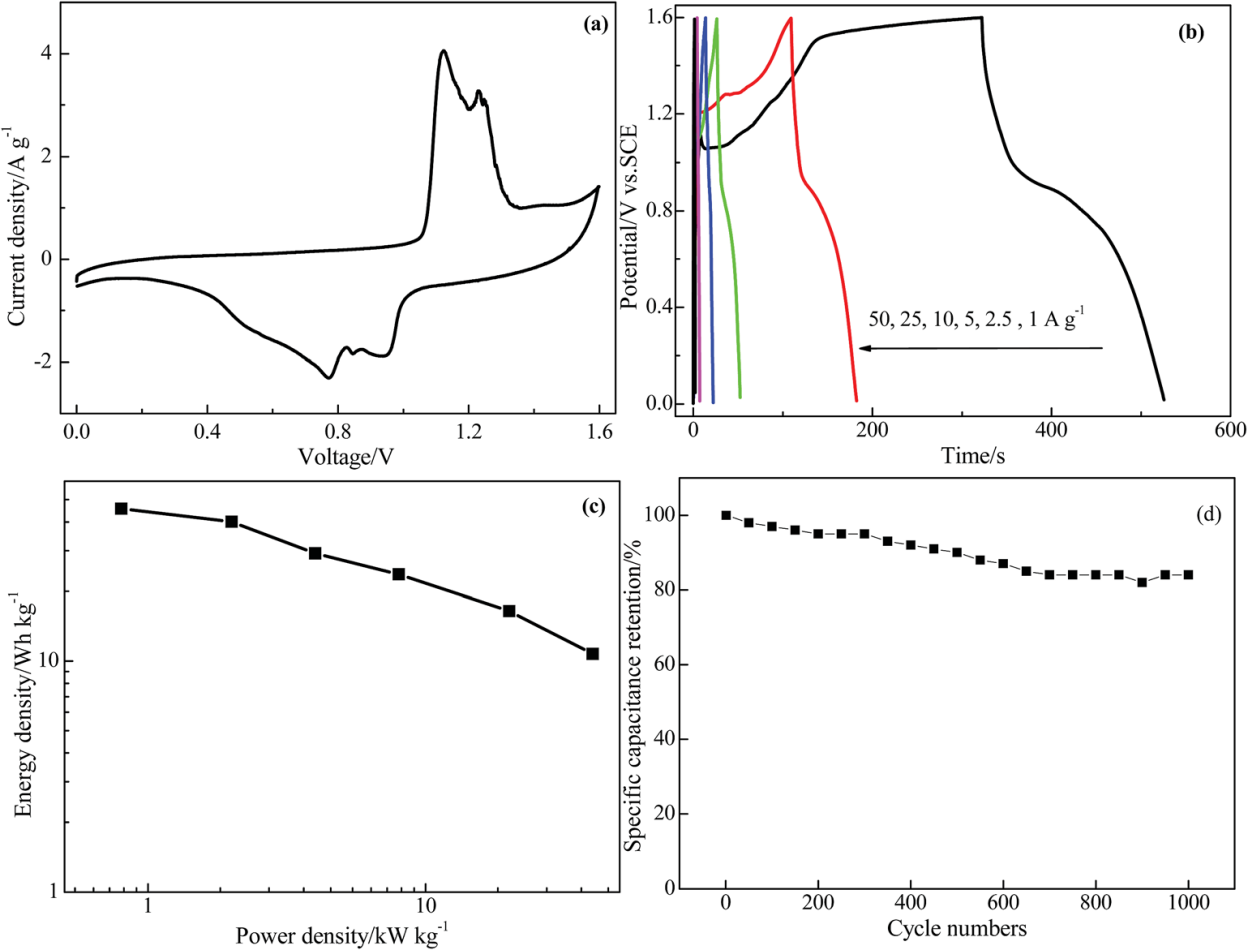
Capacitive performance of the symmetrical GR/BiVO_4_//GR/BiVO_4_ supercapacitor with a cell voltage of 1.6 V: (a) CV curve at a scan rate of 10 mV s^−1^, (b) galvanostatic charge–discharge curves at different current densities, (c) Ragone plot and (d) variation of specific capacitance retention with cycle number.

Based on the above discussion, the GR/BiVO_4_ electrode shows an extended potential window from −1.1 to 0.7 V and a high specific capacitance, and so a symmetrical supercapacitor cell was assembled to test its practical capacitive properties. In this symmetrical supercapacitor cell, equal-quality GR/BiVO_4_ free-standing composites were used as both the negative and positive electrodes. According to the test results in the three-electrode system, the GR/BiVO_4_//GR/BiVO_4_ symmetric cell should give a voltage window of 1.8 V, however, a drastic current increasing phenomenon was found when the potential was above 1.6 V, and therefore all the electrochemical tests (CV, galvanostatic charge–discharge profiling, and cycling stability) were performed in the voltage window of 1.6 V. Fig. 8a shows the CV curve of the GR/BiVO_4_//GR/BiVO_4_ symmetric cell at a scan rate of 10 mV s^−1^. Normally, no obvious redox peaks in the CV curve indicates that the charge–discharge process of the active materials is carried out at a nearly pseudo-constant rate over the whole potential window. The GR/BiVO_4_//GR/BiVO_4_ symmetrical cell exhibits a pair of evident redox peaks centred at about 0.6 and 0.7 V, resulting from the reversible oxidation and reduction of BiVO_4_ in the composite electrode.

The charge–discharge curves of the GR/BiVO_4_//GR/BiVO_4_ symmetrical supercapacitor cell are shown in Fig. 8b. All curves exhibit a distorted triangular shape, which sharply compares with that of EDLCs which have an isosceles triangular shape. The GR/BiVO_4_//GR/BiVO_4_ symmetrical supercapacitor cell shows high specific capacitance values from 128 to 21 F g^−1^ as the current densities increased from 1 to 50 A g^−1^.

The Ragone plot of the symmetrical supercapacitor cell charge–discharge process at different current densities is shown in Fig. 8c, and all of the results are based on the mass of the total active materials of the two electrodes. It can be seen that the GR/BiVO_4_//GR/BiVO_4_ symmetrical cell shows a significant energy density enhancement (45.69 W h kg^−1^) at a power density of 0.8 kW kg^−1^, which is much higher than that of a RuO_2_/graphene//RuO_2_/graphene symmetrical cell (∼11 W h kg^−1^ at 76 W kg^−1^)^33^ and a Ni@FeCo_2_O_4_@MnO_2_//Ni@FeCo_2_O_4_@MnO_2_ cell (22.2 W h kg^−1^ at 978.3 kW kg^−1^).^41^ The GR/BiVO_4_//GR/BiVO_4_ symmetrical cell, moreover, shows a higher energy density than that of some asymmetrical cells such as a GR/V_2_O_5_//GR cell (26.22 W h kg^−1^ at 425 W kg^−1^),^26^ a CoO/C//active carbon cell (30.9 W h kg^−1^ at 398 W kg^−1^),^42^ RGO-PEDOT:PSS//RGO-CNF-MnO_2_ (21 W h kg^−1^ at 471 W kg^−1^),^43^ a Co_3_O_4_//AC cell (24.2 W h kg^−1^ at 600 W kg^−1^),^44^ a NiCo_2_S_4_@PPy core–shell//activated carbon cell (43 W h kg^−1^ at 801 W kg^−1^),^45^ a MnO_2_-NHCS//NHCS cell (26.8 W h kg^−1^ at 233 W kg^−1^),^46^ NiCo_2_S_4_//active carbon (25.5 W h kg^−1^ at 334 W kg^−1^),^47^ NiCo_2_(CO_3_)_1.5_(OH)_3_@NiCo_2_S_4_//activated carbon (32.3 W h kg^−1^ at 1835 W kg^−1^)^48^ and NiCo_2_O_4_@NiO//active carbon (31.5 W h kg^−1^ at 215.2 W kg^−1^).^49^ In particular, an energy density of 10.75 W h kg^−1^ can be obtained even at a power density of 40 kW kg^−1^. The excellent capacitive performance can be ascribed to the use of an aqueous electrolyte with high ion conductivity and the synergistic effect between the GR nanosheets and BiVO_4_ particles. The GR in the composite accelerates the charge transfer through the electric double layer, while the flower-like BiVO_4_ provides a short diffusion length for the electrolyte and more electrochemically active surface for fast and reversible Faradaic reactions.

The long-term cycling performance of the symmetrical GR/BiVO_4_//GR/BiVO_4_ cell was evaluated with consecutive galvanostatic charge–discharge tests, and the experimental results are shown in Fig. 8d. After 1000 cycles at a current density of 5 A g^−1^, the specific capacitance based on the total mass of the two electrodes is about 68.9 F g^−1^, which corresponds to 84% of its initial capacitance (82 F g^−1^).

## 4. Conclusion

In this work, a free-standing GR/BiVO_4_ composite has been prepared using a one-pot hydrothermal method. The GR/BiVO_4_ shows fascinating specific capacitance values and wide potential window when used as a binder-free electrode for supercapacitors. A symmetric cell based on this GR/BiVO_4_ composite has been assembled. The symmetric supercapacitor can work in a high voltage window of 1.6 V leading to a significantly higher gravimetric energy density of 10.75 W h kg^−1^ with a high gravimetric power density of 40 kW kg^−1^. The symmetric supercapacitor, moreover, can deliver an excellent cycling stability (84% retention after 1000 cycles). This study will expand considerably the range of practical applications of BiVO_4_ and shows that it has great potential in supercapacitor applications.

## Conflicts of interest

There are no conflicts to declare.

## Supplementary Material
